# Comparisons of simulation results between passive and active fluid structure interaction models for left ventricle in hypertrophic obstructive cardiomyopathy

**DOI:** 10.1186/s12938-020-00838-4

**Published:** 2021-01-12

**Authors:** Xueying Huang, Long Deng, Heng Zuo, Chun Yang, Yunhu Song, Mary Lesperance, Dalin Tang

**Affiliations:** 1 School of Mathematical Sciences, Xiamen University, Xiamen, 361005 Fujian China; 2 Mathematical Sciences Department, Worcester Polytechnic Institute, Worcester, MA 01609 USA; 3 Department of Cardiac Surgery, Fuwai Hospital, Chinese Academy of Medical Sciences, Beijing, China; 4 School of Mathematical Sciences, Sichuan Normal University, Chengdu, Sichuan China; 5 Network Technology Research Institute, China United Network Communications Co., Ltd., Beijing, China; 6 Department of Mathematics and Statistics, University of Victoria, Victoria, BC V8P 5C2 Canada; 7 School of Biological Science and Medical Engineering, Southeast University, Nanjing, China

**Keywords:** Fluid–structure interactions, Left ventricle, Mitral valve, Systolic anterior motion, Passive computational model, Active computational model

## Abstract

**Background:**

Patient-specific active fluid–structure interactions (FSI) model is a useful approach to non-invasively investigate the hemodynamics in the heart. However, it takes a lot of effort to obtain the proper external force boundary conditions for active models, which heavily restrained the time-sensitive clinical applications of active computational models.

**Methods:**

The simulation results of 12 passive FSI models based on 6 patients’ pre-operative and post-operative CT images were compared with corresponding active models to investigate the differences in hemodynamics and cardiac mechanics between these models.

**Results:**

In comparing the passive and active models, it was found that there was no significant difference in pressure difference and shear stress on mitral valve leaflet (MVL) at the pre-SAM time point, but a significant difference was found in wall stress on the inner boundary of left ventricle (endocardium). It was also found that pressure difference on the coapted MVL and the shear stress on MVL were significantly decreased after successful surgery in both active and passive models.

**Conclusion:**

Our results suggested that the passive models may provide good approximated hemodynamic results at 5% RR interval, which is crucial for analyzing the initiation of systolic anterior motion (SAM). Comparing to active models, the passive models decrease the complexity of the modeling construction and the difficulty of convergence significantly. These findings suggest that, with proper boundary conditions and sufficient clinical data, the passive computational model may be a good substitution model for the active model to perform hemodynamic analysis of the initiation of SAM.

## Background

Hypertrophic obstructive cardiomyopathy (HOCM) is characterized by hypertrophic myocardium and obstruction in the left ventricular outflow. Patients with this disease might suffer from severe heart failure and even sudden death. Septal myectomy is the golden standard treatment for the symptomatic patients [1, 2]. However, this is a very challenging procedure as the extent of myectomy is very difficult to be determined. This is because that inadequate excision cannot abolish left ventricular outflow obstruction, while the excessive myectomy might produce ventricular septal defect or irregular heart rhythms, such as complete heart block. Therefore, a non-invasive method for helping surgeons make the optimal design of the surgery is significantly required.

Several heart models, such as structural finite element models, computational fluid dynamics models, fluid–structure interactions (FSI) models, multi-patient models, have been proposed in the literature to assess the hemodynamics and myocardial functions in heart and become increasingly important in cardiovascular research [3–13]. Cardiac tissue is generally modeled as a hyper-elastic material [14]. Heart models can be divided into passive and active models to model the myocardial behavior [8, 15–23]. The incompressible elastic solids are generally used to model soft tissue in passive models. The exponential strain-energy functions have been widely used to describe the mechanical behavior of passive myocardium [15, 18]. The Holzapfel-type energy functions were commonly used for passive tissue stress [9, 24–26]. Electrical activation is one way to trigger the cardiac active contraction [8, 16, 17, 19–21]. While this method offers much more insight into the physiological processes, it also requires patient-specific identification of all the parameters inside the electromechanical models. In our case, the left ventricle (LV) is a severely pathological LV which may differ considerably from the ones reported in literature for healthy patients. Applying time-dependent material properties in a solid model is also a method to model the active contraction [22, 27]. However, patient-specific time-dependent material properties were not able to be measured at the in vivo state due to current technological limitations. In our study, we applied an external force to implement the LV myocardial active contraction. The 3D active FSI models were applied to investigate the biomechanics of the myocardium and the intraventricular flow in LV [28]. However, it takes a lot of effort to obtain the proper external force boundary conditions to match the numerical simulation results with clinical data, which prevents the active computational models from time-sensitive clinical applications. Comparing to the active model, the pressure boundary conditions of passive models were easier to obtain. Additionally, it’s easier to obtain the convergent solutions of passive model. In this study, we constructed 12 patient-specific computed-tomography (CT)-based passive FSI models for the LV of patients with HOCM, and we compared the numerical simulation results between these passive models and their corresponding active FSI models to investigate the differences.

## Results

**Fig. 1:**
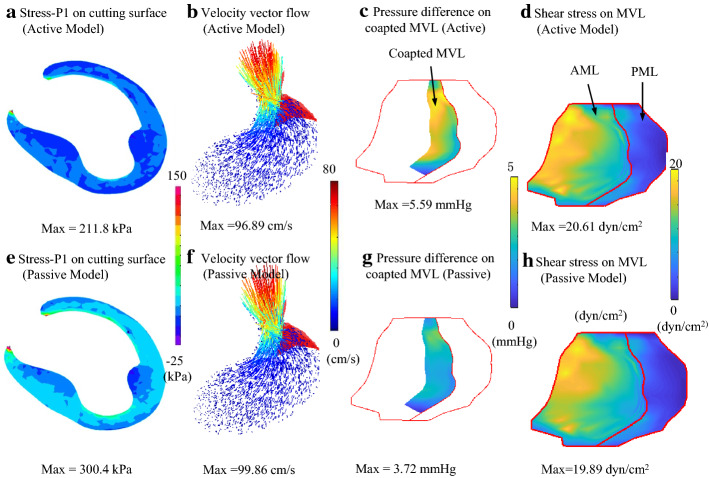
Comparisons of numerical simulation results at pre-SAM time point (5% RR interval time point) obtained from active and passive models. Active models, **a** distribution of wall stress on cutting surface; **b** 3D-velocity vector flow map; **c** distribution of pressure difference on coapted MVL; **d** distribution of shear stress on MVL; **e**–**h** were the corresponding results obtained from passive models. *AML* anterior mitral leaflet, *PML* posterior mitral leaflet

Based on 6 patients’ pre-operative and post-operative LV CT Images, 12 patient-specific active and corresponding passive FSI models were constructed in this study. Figure 1 presents the numerical simulation results of stress-P_1_ on the cutting surface, 3D velocity vector flow map, pressure difference, and shear stress distribution on the MVL at Pre-SAM time point obtained from pre-operative active and passive FSI models (patient 1). Figure 1 shows that the maximum stress value on the cutting surface obtained from passive models were 41.8% higher than that from active models, while the differences between fluid results were not significant. Details of the model comparisons are given below.

### Maximum wall stress values in passive models were 29.09% higher than those in active model

**Fig. 2:**
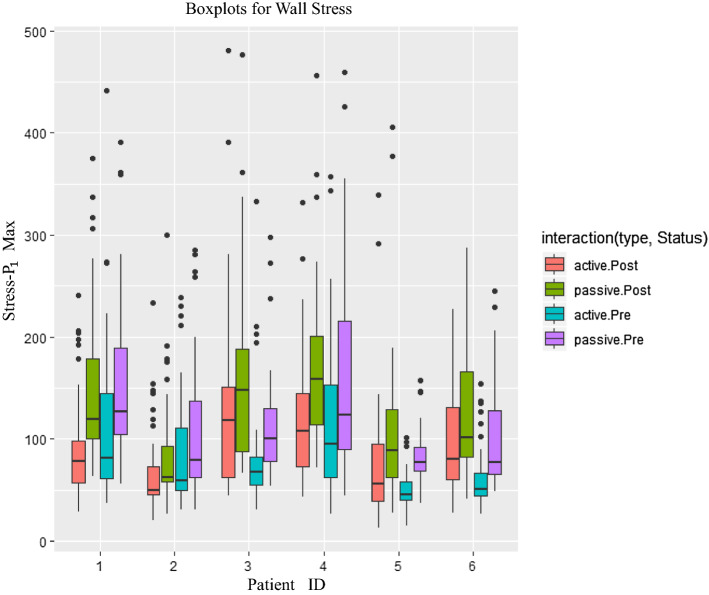
Comparisons of active and passive models in wall stress of each slice obtained from pre-operative/post-operative models for different patients. *active.Post* post-operative active model, *passive.Post* post-operative passive model, *active.Pre* pre-operative active model, *passive.Pre* pre-operative passive model. Stress-P1Max = maximum value of wall stress (stress-P_1_) values

Figure 2 presents the boxplots for the wall stress values obtained from slices in pre-operative and post-operative active and passive models. The mean values of maximum wall stress of each active model were higher than those obtained from corresponding passive model (Fig. 2).

The maximum/minimum/mean values of wall stress and wall strain of each patient obtained from active models were compared with those from passive models. The comparison results are presented in Table 1. The mean value of maximum wall stress values in passive models were found 29.09% higher than that in active models (122.7 ± 38.1 kPa vs. 95.0 ± 26.9 kPa, *p* = 0.04). No significant difference was found in maximum wall strain values between passive and active models (0.106 ± 0.015 vs. 0.112 ± 0.019, *p* = 0.39).

**Table 1:** Summary of comparisons of wall stress and strain values obtained from active and passive models

	Active (mean ± SD)	Passive (mean ± SD)	*p* value	Diff (%)
Max stress-P_1_ (kPa)	95.0 ± 26.9	122.7 ± 38.1	0.04	29.09
Min stress-P_1_ (kPa)	-33.4 ± 5.1	-34.5 ± 6.6	0.65	3.27
Mean stress-P_1_ (kPa)	2.7 ± 2.8	8.0 ± 6.0	0.01	194.85
Max strain-P_1_	0.106 ± 0.015	0.112 ± 0.019	0.39	5.73
Min strain-P_1_	0.009 ± 0.002	0.009 ± 0.001	0.50	-5.01
Mean strain-P_1_	0.046 ± 0.009	0.046 ± 0.08	0.91	0.86

Diff (%) represents the relative percentage difference based on active models

### No significant differences were found in fluid results

No significant differences between active and passive models were found in mean values of maximum pressure values and shear stress on MVL (pressure: 82.3 ± 11.7 kPa vs. 83.3 ± 12.0 kPa, *p* = 0.82; shear stress: 12.1 ± 6.8 dyn/cm^2^ vs. 11.6 ± 6.4 dyn/cm^2^, *p* = 0.85). The details of comparisons results of mean pressure and shear stress values are also listed in Table 2.

**Table 2:** Summary of comparisons of pressure and fluid shear stress (FSS) results on mitral valve leaflet between active and passive models

	Active (mean ± SD)	Passive (mean ± SD)	*p* value	Diff (%)
Max pressure (mmHg)	82.3 ± 11.7	83.3 ± 12.0	0.82	1.32
Min pressure (mmHg)	77.3 ± 11.0	80.8 ± 11.1	0.44	4.65
Mean pressure (mmHg)	80.7 ± 11.5	82.6 ± 11.8	0.70	2.33
Max FSS (dyn/cm^2^)	12.1 ± 6.8	11.6 ± 6.4	0.85	-3.82
Min FSS (dyn/cm^2^)	1.1 ± 0.3	0.8 ± 0.3	0.03	-26.61
Mean FSS (dyn/cm^2^)	5.0 ± 2.2	4.8 ± 1.9	0.55	-7.53

Diff (%) represents the relative percentage difference based on active models

### Pressure difference and shear stress on MVL were found significantly decreased after successful surgery in both of active and passive models

It was noticed that, before the surgery, the pressure on the posterior surface of MVL was higher than that on the anterior surface [28]. Therefore, to better investigate the differences between active and passive models, the pressure difference between posterior and anterior surfaces of MVL of the patients before and after surgery between different groups (patients receiving successful surgery vs. failed surgery) were investigated (Table 3). It was found that the pressure difference on MVL obtained from passive model decreased significantly after septal myectomy in satisfactory-outcome group (maximum pressure difference, 2.44 ± 1.30 mmHg vs. 0.68 ± 0.29 mmHg, *p* = 0.017; mean pressure difference, 1.17 ± 0.68 mmHg vs. 0.24 ± 0.14 mmHg, *p* = 0.024).

**Table 3:** Summary of comparisons of pre-operative and post-operative differences of maximum/mean pressure values on coapted mitral valve leaflets (MVL) and shear stress on MVL in satisfactory-outcome group (group 1, *n* = 5) and unsatisfactory-outcome group (group 2, *n* = 1) obtained from active and passive models

	Active (pre-op vs. post- op)		Passive (pre-op vs. post- op)	
	Group 1 (mean ± SD)	Group 2	Group 1 (mean ± SD)	Group 2
Pressure difference (mmHg)				
Max	4.30 ± 1.88 vs. 0.57 ± 0.36	6.65 vs. 4.39	2.44 ± 1.30 vs. 0.68 ± 0.29	4.05 vs. 2.70
Mean	2.22 ± 1.03 vs. 0.36 ± 0.16	3.05 vs. 2.02	1.17 ± 0.68 vs. 0.24 ± 0.14	1.79 vs. 1.19
Shear stress (dyn/cm^2^)				
Max	21.5 ± 4.8 vs. 10.3 ± 3.1	34.6 vs. 37	18.8 ± 2.1 vs. 9.5 ± 3.0	33.5 vs. 35.8
Mean	6.4 ± 1.8 vs. 3.3 ± 1.3	7.5 vs. 6.7	5.9 ± 1.4 vs. 3.1 ± 1.2	6.9 vs. 6.3

**Fig. 3:**
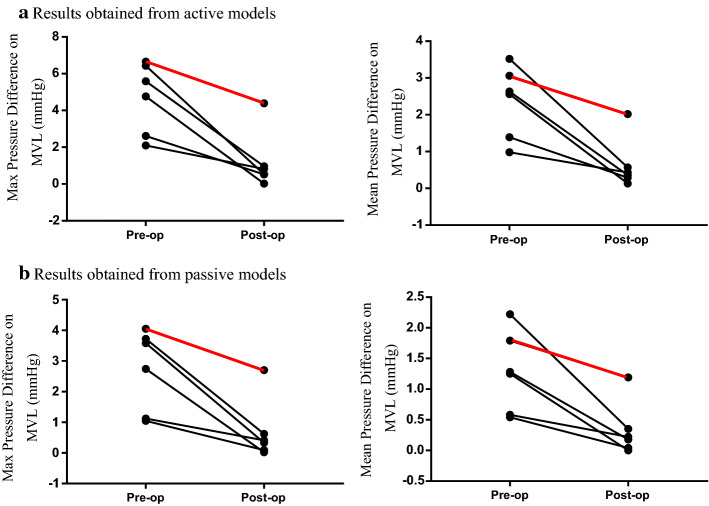
Comparisons of the difference of maximum/mean pressure values on coapted mitral valve leaflets (MVL) before (pre-op) and after surgery (post-op). The red lines indicate the patient received an unsatisfactory outcome, and the black lines indicate that the patients receiving successful surgery. **a** Results obtained from active models; **b** results obtained from passive models

Although the values of pressure differences between posterior and anterior surfaces of MVL in passive models were lower than those obtained from active models, the same observations were found from both models (Fig. 3).

**Fig. 4:**
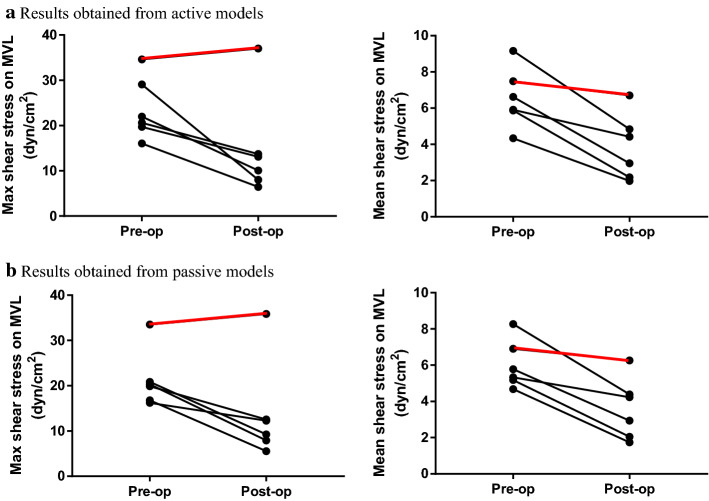
Comparisons of the mean value of maximum/mean fluid shear stress on coapted mitral valve leaflets (MVL) before and after surgery. The black lines indicate the patients with successful surgery, and the red lines indicate the patient with failed surgery. **a** Results obtained from active models; **b** results obtained from passive models

The pre-operative and post-operative shear stress on MVL obtained from active and passive models were also investigated (Fig. 4). It was observed that the max shear stress on MVL decreased 52.1% (*p* = 0.01) in active models and 49.5% (*p* = 0.004) in passive models after successful surgical septal myectomy. The mean shear stress was found decreased significantly in both of active models (pre-op: 6.38 ± 1.76 dyn/cm^2^ vs. post-op: 3.27 ± 1.30 dyn/cm^2^, *p* = 0.004) and passive models (pre-op: 5.85 ± 1.41 dyn/cm^2^ vs. post-op: 3.07 ± 1.22 dyn/cm^2^, *p* = 0.004) (Table 3). The post-operative maximum shear stress on MVL for the patient receiving failed surgery were 259% (active models, 37.0 dyn/cm^2^ vs. 10.3 dyn/cm^2^) and 277% (passive models, 35.8 dyn/cm^2^ vs. 9.5 dyn/cm^2^) higher than the mean value of those in group 1 (Table 3).

## Discussion

In this study, there were 12 active and corresponding passive FSI models constructed to compare the differences of simulation results between these two types of models. The numerical simulation results at pre-SAM time point, including wall stress and strain on the inner boundary of LV, and pressure and shear stress on MVL, were extracted to be compared. It was found that there was a significant difference in wall stress between the passive and active models. The mean value of maximum wall stress in passive models was 29.09% higher than that in active model. However, there were no significant differences of strain values and fluid results between the active and passive models. Based on simulation results obtained from passive models, the pressure difference on coapted MVL and shear stress on MVL were found decreased significantly after successful surgery, but remained still high after failed surgery. These findings are in good agreement with the results obtained from active models [28].

While the mechanisms driving the left ventricle motion in the passive model are different from real heart motion, the simulation of the hemodynamic status, the motion and deformation of LV can still be approached by the passive models. In both of our passive and active models, the boundary conditions were adjusted to match the simulation results with clinical data. That is, at pre-SAM time, the simulation results of average pressure, LV volume, and LVOT velocity were matched with clinical measured data well in both of passive and active models. Our results indicated that, if we focus on the simulation results of left ventricle at some specific time points, the passive model may provide simulation results of blood flow matching well with active models with proper boundary conditions and sufficient clinical data.

Table 4 presents the number of manually adjustments of simulation experiments for passive model and active models. The total number of numerical simulation experiments for active model to receive the proper boundary conditions was about two times more than that of passive models ($15.8\pm 6.6$ vs. $5.2\pm 1.6$). Every simulation experiment takes around 3 h. Therefore, for each FSI model, it took at least more than 30 h to obtain the proper boundary conditions for active models comparing to passive models. Suppose the researcher works 6 h/day, 5 weekday/week. Which means the patient needs around one more week to receive the simulation results and surgical plan. In addition, to obtain the convergent solutions, for every simulations, the active models needs 85% more iteration times than passive models (active vs. passive, 12.3 $\pm 3.3$ vs. 6.7 $\pm 0.7$). The passive models also decrease the difficulty of convergence significantly. Since the goal of our study was to perform hemodynamic analysis of LV to investigate the mechanisms of SAM, and the passive models provided good approximated hemodynamic simulations, the passive models may be used as a good substitution model to active models to investigate the initiation of SAM.

**Table 4:** Summary of comparisons of the total number of numerical experiments for obtaining the proper boundary conditions between active models and passive models

Model	Passive	Active
P1-pre	7	14
P1-post	1	3
P2-pre	7	24
P2-post	4	12
P3-pre	6	13
P3-post	4	15
P4-pre	5	28
P4-post	5	13
P5-pre	7	25
P5-post	5	14
P6-pre	5	17
P6-post	6	12
Mean $\pm$ sd	$5.2\pm 1.6$	$15.8\pm 6.6$

Some limitations of this study are acknowledged here. The main limitation of the study is the small sample size which results in limited statistical power. The reason for the small sample size is that the computational modeling method takes large time costs, (1) it takes a lot of effort to find the proper boundary conditions for active models; (2) the construction procedure for both of active and passive models also takes time; (3) it is not easy to obtain patient-specific CT data, especially for the follow up CT data. Currently, multi-patient studies for heart model simulations is still rare. Adding more patients will be our future effort. Applying commercial software, such as HyperMesh, to automatically create the finite element mesh to save the construction time will be considered. Although CT images provided high-resolution medical images, the chordae tendineae is still impossible to be displayed. Therefore, the chordae tendineae is not included in our model. Due to rapid motion, our technique also restricts us from simulating mitral valve dynamic motions. From the end of the isovolumic systole to the pre-SAM time point, the mitral valve is almost closed, therefore, this simplification would have little impact on fluid results for analyzing the mechanisms of initiation of SAM. It should also be noted that, the fibre orientation plays a critical role both in passive inflation [26, 29, 30] and active contraction [31, 32]. The anisotropic models may improve the computational prediction accuracies. However, in the heart in HCM patients, the alignment of muscle cells or myocardial disarray was found very irregular and disorganized [33, 34]. The fiber orientation data were not able to be obtained with current technology. Therefore, we assumed that the material of the left ventricle was isotropic. In the future, the single or multi-layer anisotropic models would be introduced into our model to investigate other patients’ left ventricle models.

## Conclusion

In this study, based on CT images of 6 HOCM patients before and after surgical septal myectomy, 12 active and corresponding passive FSI models were developed to compare the simulated biomechanics and hemodynamics behaviors between passive and active models. It was found that, between these models, there was a significant difference in wall stress, but the differences in hemodynamic simulation results were not significant. Compared with active models, the passive models decrease the complexity of the modeling construction and the difficulty of convergence significantly. Therefore, with proper boundary condition, the passive model may be a good approximation to active model with less computational cost to perform hemodynamic analysis for left ventricle to investigate the initiation of SAM. Prospective and large-scale studies are needed to further validate our findings.

## Methods

### Data acquisition

Institution review board approval on human subject research at Fuwai Hospital was obtained for this study. For model constructions and analysis, the CT images before and after surgery were obtained from six patients with HOCM who received septal myectomy in Fuwai Hospital. All these six patients had severe systolic anterior motion (SAM) before surgery. Based on the outcomes of surgery, patients were categorized into two groups, satisfactory-outcome group (patient no. 1–5) which successfully eliminated SAM and mitral regurgitation, and unsatisfactory-outcome group (patient no. 6) which had residual SAM and mitral regurgitation after surgery. Pre-operative and post-operative echocardiographic characteristics are summarized in Table 5.

**Table 5:** Echocardiographic characteristics for patients

Patient no.	Gender	Age	Anterior septal		SAM and		LVOTG,		Mitral regurgitation	
			Thickness (mm)		beginning time		mm Hg			
			Pre-op	Post-op	Pre-op	Post-op	Pre-op	Post-op	Pre-op	Post-op
1	Male	18	39	30	+, 6%RR	–	55	16	–	–
2	Male	52	21	14	+, 5%RR	–	104	7	Moderate	–
3	Male	47	34	22	+, 5%RR	–	100	7	Moderate	Trace
4	Female	54	40	30	+, 6%RR	–	81	20	Moderate	–
5	Male	55	27	19	+, 8%RR	–	100	6	Moderate to severe	–
6	Male	54	24	15	+, 8%RR	+, 8%RR	81	64	Moderate	Mild

*LVOTG* left ventricular outflow tract gradient, *pre-op* pre-operation, *post-op* post-operation, *SAM* systolic anterior motion

Cardiac CT images were obtained at every 5% inter-beat (RR) interval. In all these six patients, the beginning time of SAM ranged from 5 to 8% RR interval in the cardiac cycle. Therefore, to investigate the initiation of SAM, the pre-operative and post-operative CT images at 5% RR interval were selected as pre-SAM time point data to construct the computational models. The field of view was 256 mm × 256 mm, the matrix was 512 × 512, and the slice thickness was 0.625 mm. The original slice thickness of the CT images was 0.625 mm, and there were about 110–150 slices of images covering the LV. The segmentation was performed manually by Dr. Deng to obtain digital contours of each component for modeling constructions. The segmentation results were all examined by experienced radiologist. Volume component-fitting method was employed to generate meshes for left ventricle with irregular geometry. Using this technique, both the left ventricle and fluid domains were divided into thousands of small “volumes” to curve-fit the geometry. The edge of each volume will be further divided into several divisions to generate the final mesh in ADINA (ADINA R&D, Watertown, MA). By applying this method, the mesh generated would not be too distorted under large deformation. More details about the CT images and segmentation for the construction of FSI models can be found from Deng et al. [28, 35]. Patients’ heart rate and blood pressure at the time of CT examinations were used in the simulation. The patient-specific LV volume, pressure and the left ventricular outflow tract (LVOT) velocity at pre-SAM time obtained from echo and MRI data were used to verify the simulation results (Table 6).

**Table 6:** Summary of the comparisons of simulation results of LV pressure, volume, and the left ventricle outflow tract (LVOT) velocity at pre-SAM time obtained from active (Act) and passive (Pas) models, and those from clinical data

Patient no.	LV pressure (mm Hg)			LV volume (ml)			LVOT velocity (m/s)		
	Clinical data	Simulation results		Clinical data	Simulation results		Clinical data	Simulation results	
		Act	Pas		Act	Pas		Act	Pas
1	67	65.9	68.3	136.7	137	137	0.56	0.57	0.57
2	85	85.2	87.9	128.6	128	129	1.00	0.97	0.99
3	85	85.5	87.1	126.5	125	127	0.83	0.79	0.83
4	75	73.7	80.5	146.4	147	147	1.20	1.19	1.20
5	72	72.2	76.0	191.5	191	192	1.10	1.11	1.10
6	93	90.7	99.5	106.5	106	107	1.36	1.35	1.32

### Solid models

The modified non-linear Mooney–Rivlin model was adopted to characterize the mechanical behavior of LV myocardium. The strain-energy function for the isotopic incompressible Mooney–Rivlin model was expressed as,

1 $$W={c}_{1}\left({I}_{1}-3\right)+{c}_{2}\left({I}_{2}-3\right)+{D}_{1}{[\mathrm{e}}^{{D}_{2}({I}_{1}-3)}-1],$$

where ${I}_{1}$, and ${I}_{2}$ are the first and second strain invariants,

2 $${I}_{1}=\sum {C}_{ii},{I}_{2}=\frac{1}{2}({I}_{1}^{2}-{C}_{ij}{C}_{ij})$$

${\varvec{C}}=\left[{C}_{ij}\right]={{\varvec{X}}}^{\mathrm{T}}{\varvec{X}}$ is the right Cauchy-Green deformation tensor, ${\varvec{X}}=\left[{X}_{ij}\right]=[\partial {x}_{i}/\partial {a}_{j}]$, where ${x}_{i}$ is the current position, ${a}_{j}$ is the original position, and ${c}_{1}$,${c}_{2}$, ${D}_{1}$, and ${D}_{2}$ are material constants [27, 36]. Details of the determination of the patient-specific material constants have been described in our previous publication [28, 35].

### Fluid dynamics simulation

The blood in the LV was treated as a laminar, Newtonian, viscous and an incompressible fluid. In this study, we set the viscosity of $\mu =0.04\mathrm{ dyn }{\mathrm{cm}}^{-2}$ and density of $\rho =1\mathrm{ g }{\mathrm{cm}}^{-3}$ for blood properties. The Navier–Stokes equation with Arbitrary Lagrangian Eulerian formula was used as the governing equation. Boundary conditions in the simulation were set such that, during the ejection phase, when blood was ejected out of the left ventricle, the outlet (aortic valve) kept open and the inlet (mitral valve) was closed (flow velocity was set to zero, and the pressure was left unspecified). A no-slip boundary condition between the interfaces was assumed. The structure and fluid models were coupled through their interfaces [6, 27, 36]. The complete fluid model is given by,

3 $$\uprho \left(\partial {\varvec{u}}/\partial t+\left(\left({\varvec{u}}-{{\varvec{u}}}_{g}\right)\bullet \nabla \right){\varvec{u}}\right)=\nabla p+\mu {\nabla }^{2}{\varvec{u}}$$

4 $$\nabla \bullet {\varvec{u}}=0$$

5 $${\varvec{u}}{|}_{\Gamma }=\frac{\partial {\varvec{x}}}{\partial \mathrm{t}},{\frac{\partial \mathbf{v}}{\partial n}|}_{\mathrm{inlet},\mathrm{ outlet}}=0$$

6 $${p|}_{\mathrm{valve}}={p}_{\mathrm{inlet}}\left(t\right), {{\varvec{u}}|}_{\mathrm{aorta}}=0\; (\mathrm{filling} \mathrm{phase})$$

7 $${p|}_{\mathrm{aorta}}={p}_{\mathrm{outlet}}\left(t\right), {{\varvec{u}}|}_{\mathrm{valve}}=0\;\boldsymbol{ } (\mathrm{ejection phase})$$

8 $${\sigma }_{ij}^{f}{n}_{j}^{f}{|}_{\mathrm{interface}}={\sigma }_{ij}^{s}{n}_{j}^{s}{|}_{\mathrm{interface}}$$

where ${\varvec{u}}$ and $p$ are fluid velocity and pressure, ${{\varvec{u}}}_{g}$ is mesh velocity, $\Gamma$ stands for the left ventricle inner wall, ${\varvec{\sigma}}$ is structure stress tensor, (superscripts, *f* and *s*, indicate blood and the left ventricle, respectively), and ${\varvec{n}}$ is the outward normal directions. The governing equations of (Eqs. (3–4)) would be rewritten to weak form by using Galerkin method [36]. Then, interpolation functions would be introduced for each element, and finite element governing equations are expressed in terms of interpolation functions. Difference method and Newton–Raphson iteration method were also used to obtain the solution iteratively.

### Solution method

In the construction process of both of active and passive models, the initial shrinkage rate was set as 5% to receive the zero-load geometry which is the starting geometry for the numerical simulation [37, 38]. When the pressure was applied at inlet, the left ventricle expanded in short-axis and long-axis direction, and then regained its in vivo morphology. During this phase, the mitral valve (MV) kept open and the blood filled into LV though MV. At the end of this phase, LV reaches its maximum volume and the pressure in LV increases to end of isovolumic systole pressure. The information of the blood flow and mechanics of LV were received as the starting state of the simulation for the phase from end of systole phase to pre-SAM time point. The details of the pre-shrink process can be found from our previously published paper [28, 35].

The finite element meshes was generated by the volume component-fitting method [6, 27]. The constructed computational models were solved by commercial software ADINA (ADINA R&D, Watertown, MA), which uses the Newton–Raphson iteration and unstructured finite elements method. The iterative FSI coupling solution method, which requires less memory than the Direct FSI Coupling method, was applied to handle fluid–structure interaction. At each time step, the fluid and solid equations will be solved individually where the latest information provided by the other part is used as boundary conditions at each time step. Specifically speaking, the solid model firstly will be solved with the latest pressure and stress condition provided by the flow part of the last time step. Then, the fluid model will be solved by using the displacement and velocity obtained from the structure part as the boundary conditions. These two steps will be repeated until the convergence is reached. Mesh analysis was performed for each model by reducing the mesh density in each dimension by 10% until differences between solutions from two consecutive meshes were negligible (less than 1% in L2-norm). The optimal element size in each dimension is between 0.05 and 0.1 cm. For each patient, there were around 90,000 elements and 60,000 elements for the solid and fluid model, respectively.

### Differences between the passive and active models

The simulations of the phase from end of systole phase to pre-SAM time point between active model and passive model were different. In passive models, the left ventricle muscle was deflated by the patient-specific pressure condition which was scaled based on measured blood pressure conditions, and the pressure difference between LV and aorta which was obtained from ultrasound scanning. In active models, we simulated the LV active contraction by specifying the external forces on epicardium, and the pressure condition at outlet (aortic valve) from the end of isovolumic systole phase to pre-SAM time point (5% RR) [28]. Based on the clinical measured data of LV volume, LV pressure, and LVOT velocity obtained from echo and MRI data at the end of isovolumic systole phase and pre-SAM time point, the external forces were applied on each element of the epicardium, and the pressure condition at outlet at each time step were numerically interpolated. The boundary conditions were manually adjusted accordingly, such that the difference between clinical measured data and the simulation results were less than 5%. The real LV active contraction motion was then implemented. The adjustment of the boundary conditions was primarily based on the comparisons of numerical results and clinical data at each time step. For more details about the construction of active and passive models, we refer the reader to our previous publications [28].

### Data extraction and statistical analysis

The stress, strain, and shear stress are all tensors, therefore, the maximum principal stress (stress-P_1_), maximum principal strain (strain-P_1_), and maximum shear stress at each node were chosen as wall stress, strain, and shear stress, respectively, for convenience. Data for wall stress and wall strain of all integral nodes on the inner boundary of left ventricle (endocardium) and fluid shear stress and pressure of all integral nodes on mitral valve were extracted from 3D FSI solutions. For each slice, the maximum/mean/minimum values of the simulation results of wall stress, wall strain, fluid shear stress, and pressure were selected for analysis. Which means, there were at least 20 samples come from the same patient (but comes from different slice). The data were in non-independent data structure. Therefore, the linear mixed-effect-model [39, 40] was used to compare the simulation results between passive and active models. The statistical significance was established at a *p* value of < 0.05. All statistical analyses in this study were conducted using R software (version 3.5.1).

## Data Availability

The datasets generated during and/or analyzed during the current study are available from the corresponding author at reasonable request.

## Abbreviations

FSI: Fluid–structure interactions
MVL: Mitral valve leaflet
SAM: Systolic anterior motion
HOCM: Hypertrophic obstructive cardiomyopathy
LV: Left ventricle
CT: Computed-tomography
RR: Inter-beat
LVOT: Left ventricular outflow tract
MV: Mitral valve
Stress-P_1_: Maximum principal stress
Strain-P_1_: Maximum principal strain
